# Understanding the intention of consumers towards 3D food printing: exploratory study of psychological factors and sensory analysis

**DOI:** 10.1007/s13197-025-06261-8

**Published:** 2025-03-18

**Authors:** Mohammed A. Bareen, Sangeeta Prakash, Jatindra K. Sahu, Bhesh Bhandari, Satyanarayan Naik

**Affiliations:** 1 University of Queensland– IIT Delhi Academy of Research (UQIDAR), New Delhi, 110016 India; 2 Food Customization and Research Lab, Centre for Rural Development and Technology, Indian Institute of Technology Delhi, New Delhi, 110016 India; 3 School of Agriculture and Food Sustainability, The University of Queensland, Brisbane, QLD 4072 Australia

**Keywords:** Structural equation modeling, Confirmatory composite analysis, Consumer demographics, Simple random sampling, Infill pattern

## Abstract

This study examines consumer acceptance and behavioral intentions towards 3D food printing (3DFP) technology for household use, shedding light on factors influencing its market adoption. Integrating constructural level theory with novel product acceptance research, the study identifies satisfaction as a critical mediator between prior knowledge of 3D food printing, consumer innovativeness & adaptability, and behavioral intentions. Prior knowledge of 3D food printing and consumer innovativeness and adaptability significantly predict behavioral intentions (β = 0.807* and β = 0.605*, respectively), with satisfaction also playing a direct positive role (β = 0.366*). Conversely, Novel Technology Neophobia negatively impacts behavioral intentions (β = −0.027). Satisfaction mediates the effects of prior knowledge of 3D food printing and consumer innovativeness & adaptability on behavioral intentions (β = 0.406* and β = 0.200*). A sensory evaluation by a semi-trained panel (*n* = 29) reveals that 3D printed food samples differ significantly (*p* < 0.05) in appearance and cohesiveness, particularly with varied infill densities. The findings highlight the importance of understanding consumer perceptions and the role of satisfaction in promoting 3DFP adoption, especially for customized traditional milk sweets in domestic settings. This research underlines the need for targeted communication strategies to emphasize the personalization benefits of 3DFP in home use.

**Supplementary Information:**

The online version contains supplementary material available at 10.1007/s13197-025-06261-8.

## Highlights

- Innovative focus on consumer attitudes toward 3D food printing (3DFP).
- Examination of psychological and sensorial factors in 3DFP adoption.
- Integration of structural equation modeling in consumer perception study.

**Supplementary Information:**

The online version contains supplementary material available at 10.1007/s13197-025-06261-8.

## Introduction

Global challenges like greenhouse gas emissions, food waste, and food insecurity are disrupting traditional food manufacturing, necessitating an adaptable system that merges conventional methods with advanced technologies (Brunner et al. 2018). 3D food printing (3DFP) emerges as a promising solution, offering efficiency, cost-effectiveness, unique food combinations, and sustainability (Demei et al. 2022). While its technical aspects are well-documented, consumer attitudes and acceptance receive less attention. Traditional methods often fall short in predicting consumer adoption of newer technologies, making investments in this emerging technology risky (Van Kleef et al. 2005). Understanding consumer perceptions, especially in the context of food innovations, is crucial. In particular, it is required to know (a) the product to which the innovation is applied and (b) which customer perceptions are most important in the context of food innovations and how they interact to produce an overall reaction. The inability to directly quantify impressions and decisions which are in the minds of consumers-complicates both of these objectives.

Concerns about food safety, palatability, and nutritional content are significant barriers to 3DFP adoption (Ross et al. 2022). Initially seen as beneficial in non-domestic settings like military or emergency aid, 3DFP is increasingly viewed as viable for household use. However, research gaps exist regarding general consumer acceptance, with prior studies focusing on extreme user groups like astronauts or chefs (Burke-Shyne et al. 2020; Gayler et al. 2018) or kitchen personnel. In India, milk sweets, a staple, present a unique opportunity for 3DFP. Its precision and efficiency can revolutionize their production, a market dominated by small businesses facing hygiene and quality concerns (Ahmed et al. 2020).

The current study addresses the lack of research on the factors influencing 3DFP for household use. In contrast to other studies on the consumer acceptance of 3DFP, a hypothesized correlation was looked on between the factors that drive behavioural intentions’ using a structural equation modeling (SEM) based on the partial least square (PLS) approach. It aims to guide global manufacturers in developing 3D food printers that meet consumer expectations, transitioning from novelty to practical household appliances.

## Theoretical framework and hypothesis development

This section delves into the key factors expected to influence domestic consumer adoption of the 3DFP technology, drawing on relevant literature and forecasts. At the forefront, the prediction model for consumer adoption of a 3DFP primarily focuses on three core elements: their product knowledge (Brunner et al. 2018), inherent innovativeness (Lynn and Harris 1997), and perception of the product’s novelty (Hoeffler 2003). We build upon constructural level theory, which explores the contrasting ways in which individuals mentally process the appeal and practicality of actions in the present versus the future (Van Kleef et al. 2005). This framework is crucial in understanding how consumers weigh their desire for satisfaction against the apprehension of novel technology, particularly in the context of new products or services. Our study constructs hypotheses to uncover both the direct and indirect relationships among these variables, aiming to develop a comprehensive model that explains consumer behavioral intentions in adopting 3DFP for home use.

### Behavioural intentions

The term ‘behavioural intention’ is crucial in understanding consumer behavior, especially regarding novel foods. It refers to an individual’s willingness to pursue a goal, often linked to product repurchase or recommendation likelihood (Lee et al. 2018). Key influences on this intention include personal control, attitude towards behavior, and perceived social norms.

This concept is particularly relevant in studying reactions to new foods like algae, seaweed, and cultured meat, as explored by researchers including Gajaria and Mantri (2021), and others. For 3DFP, behavioral intentions significantly impact consumer acceptance. Those with positive attitudes towards technology and new foods are more likely to accept 3DFP, while those with negative views or reluctance towards new foods are less inclined. This understanding is vital for the successful marketing of novel food technologies.

### Previous knowledge of 3D food printing

Knowledge significantly influences self-efficacy, which is the belief in one’s ability to control specific behaviors. This concept is crucial in assessing perceived behavioral control and intentions, as Tsai et al. (2021) affirm, particularly in challenging scenarios similar to unfamiliar food choices. Research, including Brunner et al. (2018), shows that both perceived and factual knowledge impact attitudes toward new food technology, like 3DFP. Based on this evidence, it can be reasonably inferred that there is an extension of the positive relationship between knowledge of 3DFP and consumer’s behavioural intentions, as suggested in the following hypothesis:

#### H1

*Prior knowledge of 3D food printing*,* particularly understanding its role in food customization and personalized nutrition*,* is directly and positively correlated with consumers’ behavioral intentions to adopt 3DFP as a kitchen appliance.*

### **Consumer innovativeness** & **adaptation behavior**

Consumer innovativeness denotes the level at which people are willing to experiment with new products and technologies. Individuals with a high level of trust are less inclined to act defensively (i.e., are less risk-averse) and more willing to pay the total price for new foods as identified by Siddiqui et al. (2022a, b). It has been established that market strategists can use the consumers’ inclination towards newness to predict if they will adopt new technology and that consumers’ tendency for innovation has a direct and positive effect on the adoption of innovative behavior. 3DFP is a unique, creative, and disruptive food technology that may attract early adopters and innovators who aspire to be the first among their peers to experience the most cutting-edge culinary innovation.

Therefore, it is suggested that:

#### H2

*Consumer innovativeness*,* specifically in exploring new food preparation technologies like 3DFP that offer customized and intricate food designs*,* is directly and positively correlated with behavioral intentions to use 3DFP as a kitchen appliance.*

### Novel technology neophobia

Technological advancements in food production, often met with consumer skepticism known as novel technology neophobia, pose challenges for industry adoption. The food technology neophobia scale by Cox and Evans (2008) is a key tool in measuring this resistance, focusing on technology’s impact on consumer acceptance. This psychological factor particularly affects innovations like 3DFP, with consumers wary of their unfamiliarity and uncertain health impacts. Studies, including Lupton and Turner (2017) and Brunner et al. (2018) indicate persistent skepticism towards 3DFP, even with increased information.

Therefore, it is proposed that:

#### H3

*Novel technology neophobia*,* specifically related to the perceived complexity and novelty of 3D food printing processes*,* is directly and negatively correlated with consumers’ behavioral intentions to use 3DFP as a kitchen appliance.*

### Satisfaction

Satisfaction, as defined by Homburg et al. (2002) is a consumer’s cognitive and affective evaluation, comparing a product or service’s performance against expectations. When this performance meets or exceeds expectations, satisfaction occurs; otherwise, dissatisfaction arises. Recent studies highlight satisfaction’s role in shaping consumer attitudes towards novel food technologies. For instance, Baker et al. (2022) found that satisfaction with functional foods and ingredients enhances purchase likelihood. Siddiqui et al. (2022b) linked satisfaction with edible packaging and lab-grown meat to increased purchase and consumption intentions. These studies collectively suggest that satisfaction significantly influences consumer acceptance of innovative food technologies, driving their market success. By extending the aforementioned findings to the 3DFP context, the following hypothesis is formulated:

#### H4

*Consumer satisfaction*,* particularly with the quality*,* customization*,* and novelty of foods produced by 3DFP*,* is directly and positively correlated with behavioral intentions to use 3DFP as a kitchen appliance.*

Given the importance of customer satisfaction in fostering loyalty and maintaining long-term connections, previous studies have focused on its antecedents to pinpoint areas for development. Therefore, food quality and characteristics, along with consumer knowledge, and satisfaction have become focal points in food industry research. Previous studies have shown a positive correlation between knowledge and acceptance, as information can serve as the foundation for making a decision, leading to a more satisfying outcome for consumers when they are well informed about a particular concern. Since 3DFP is realized as a novel alternative to traditional food items, it is anticipated that customer satisfaction may mitigate the relationship between innovativeness and the behavioural intentions to use 3DFP as a kitchen appliance. All these observations can be accounted for the following hypotheses:

#### H5

*Knowledge of 3D food printing*,* specifically regarding its capabilities in creating personalized*,* nutritious*,* and visually appealing foods*,* is directly and positively correlated with consumer satisfaction in using 3DFP as a kitchen appliance.*

#### H6

*Consumer innovation*,* particularly in leveraging 3DFP to explore creative and health-conscious food options*,* is directly and positively correlated with customer satisfaction in using 3DFP as a kitchen appliance.*

#### H7

*Satisfaction with 3DFP mediates the relationship between (H7a) knowledge of 3D food printing*,* including its specific applications in food personalization*,* and consumer behavioral intentions; and (H7b) consumer innovation in utilizing 3DFP for novel food experiences and consumers’ behavioral intentions to use 3DFP as a kitchen appliance.*

## Materials and methods

### Samples

The semi-solid formulations of heat acid coagulated milk (HACM) with whey protein isolate (WPI) and maltitol (MT) were produced using a method described by Bareen et al. (2021). The process included heating skim milk to 80 ºC for 30 min and then coagulating it with a 1.62% citric acid solution at 70 ºC. WPI (4% w/w) and MT (2% w/w) powders were added to the HACM semi-solids at room temperature to maintain a total solid content of 40% (w/w) in the formulations. To make HACMP composites, 36% (w/w) of MT was added to the HACM and then mixed. This investigation employed an extrusion-based 3D food printer (Type E, Createbot, Shiyin Co Ltd, Hangzhou, China) with a 35 mL syringe capacity. The process of making the 3D printing ink and the printer set-up used for this investigation is illustrated in Fig. S1. The formulations were then homogenized at 500 rpm for 5 min to ensure proper mixing. The samples were then cooked at 80ºC while constantly stirring for 15 min until the moisture content decreased to 30% (w/w). The cooked formulations were left at room temperature for 2 h to reach equilibrium before undergoing sensory evaluation on the same day.

### Measurement procedure

#### Data collection

A web-based self-administered questionnaire (SAQ) was designed to elicit information about consumers’ perspectives on the 3D printing of milk sweets. 277 Indian consumers were questioned between November 2022 and January 2023 using the web-based SAQ. The first section of the survey inquired into the respondent’s demographics. On the next page, the motivation of the study and background of 3DFP technology was briefed. Several images and a video demonstrating 3D-printed and commercially available milk sweets without printing were also included to show customers the practical use of the research. An Ex-ante power analysis was conducted using G* Power software with a medium effect size of 0.15, a significance level of 0.05, and five predictors arising from the conceptual model under investigation suggested that a minimum of 138 participants was necessary to reach 0.95 power (Faul et al. 2009). Hence, the sample size was large enough to obtain reliable results. After removing the surveys with blanks from the original dataset, 277 were usable for analysis. The demographic information of the respondents is shown in Table S1.

#### Participants and procedures for sensory trails

A sensory testing was conducted to understand how well Indian consumers would receive 3D-printed milk sweets. The formal assessment involved ranking test in which the participants were asked to rate their preference between two different 3D-printed samples, one with a rectilinear infill pattern at 50% infill and another with at 70% infill, both of which were 30 mm in diameter and 15 mm in height. The samples were served in a randomized order, each with a different three-digit code. The sensory test was conducted in a sensory analysis laboratory using RedJade sensory evaluation software (RedJade Sensory Solutions LLC, CA, USA) at “Removed for blind peer review”. Twenty-nine (14 female, 15 male) Indian consumers from “Removed for blind peer review”, participated in the sensory test. The study was conducted according to the guidelines of “Removed for blind peer review”. Moreover, each participant read and signed an informed consent declaration before taking the test.

### Measurement design

#### Construction of latent variables

The dependent latent variable (LV) is behavioural intention (BI), while the independent LVs are novel food technology neophobia (NFTN), knowledge of 3D food printing (3DPK), and consumer innovativeness & adaptation behavior (CIAB). Satisfaction (SAT) is the mediator. All questionnaire items were derived from, and with minor modifications applied, pre-existing and tested scales to ensure the content validity of the rankings. The novel food technology neophobia study utilized the research of Cox and Evans (2008), satisfaction, a significant LV in the study, the multi-factor definition presented by Lee et al. (2018) was used. The emotional perspective of latent variable was adopted in this conception. All latent variables in the model were conceptualized and measured as reflective variables. All latent variables were measured using a Likert scale of agreement from 1 to 5 (strongly disagree to strongly agree). To investigate the potential impact of confounding factors on the study’s conclusions, the respondents’ gender, age, and level of education were used as control variables.

#### Descriptive test design for sensory analysis

The sensory evaluation procedure was a generic descriptive analysis, including aspects of the quantitative descriptive analysis and spectrum approaches. The sensory variables evaluated were appearance, hardness, cohesion, and smoothness on a 9-point hedonic scale (1 = none/very slightly to 9 = extremely). The preference of each attribute was evaluated on a 5-point hedonic scale (1 = extremely dislike to 5 = extremely like), excluding the preference of overall appearance, a method previously used by Dery et al. (2021). At the end of each test, the semi-trained panelist had to choose which sample they preferred the most. After each question, the participants were also asked to comment on the chosen response.

### Data analysis

For empirical verification of the theoretical model, a Partial least square (PLS) -Structural Equation Modeling (SEM) procedure was used and implemented in Smart PLS 4. This is because testing indicated that the data was non-normal, and in such cases, Smart PLS is recommended over covariance-based (CV) SEM (Becker et al. 2022). For this investigation, the Kolmogorov-Smirnov multivariate normality test was used using SPSS software (SPSS Science Inc, Illinois) to establish that the data did not follow a multivariate normal distribution. In addition, PLS was employed as an estimation approach since it can estimate complicated models with little data needs. It is highly recommended for preliminary and exploratory investigations of new model developments, such as the one conducted in this study, which involved testing a new, not-yet-empirically validated model.

The sensory profiling data were statistically assessed using the SPSS program, with a significance threshold of α = 0.05, using one-way factorial analysis of variance (ANOVA) and Scheffe’s post hoc analyses. The results are expressed as arithmetic mean (x) ± standard deviation (SD).

## Results

### Measurement model

**Fig. 1:**
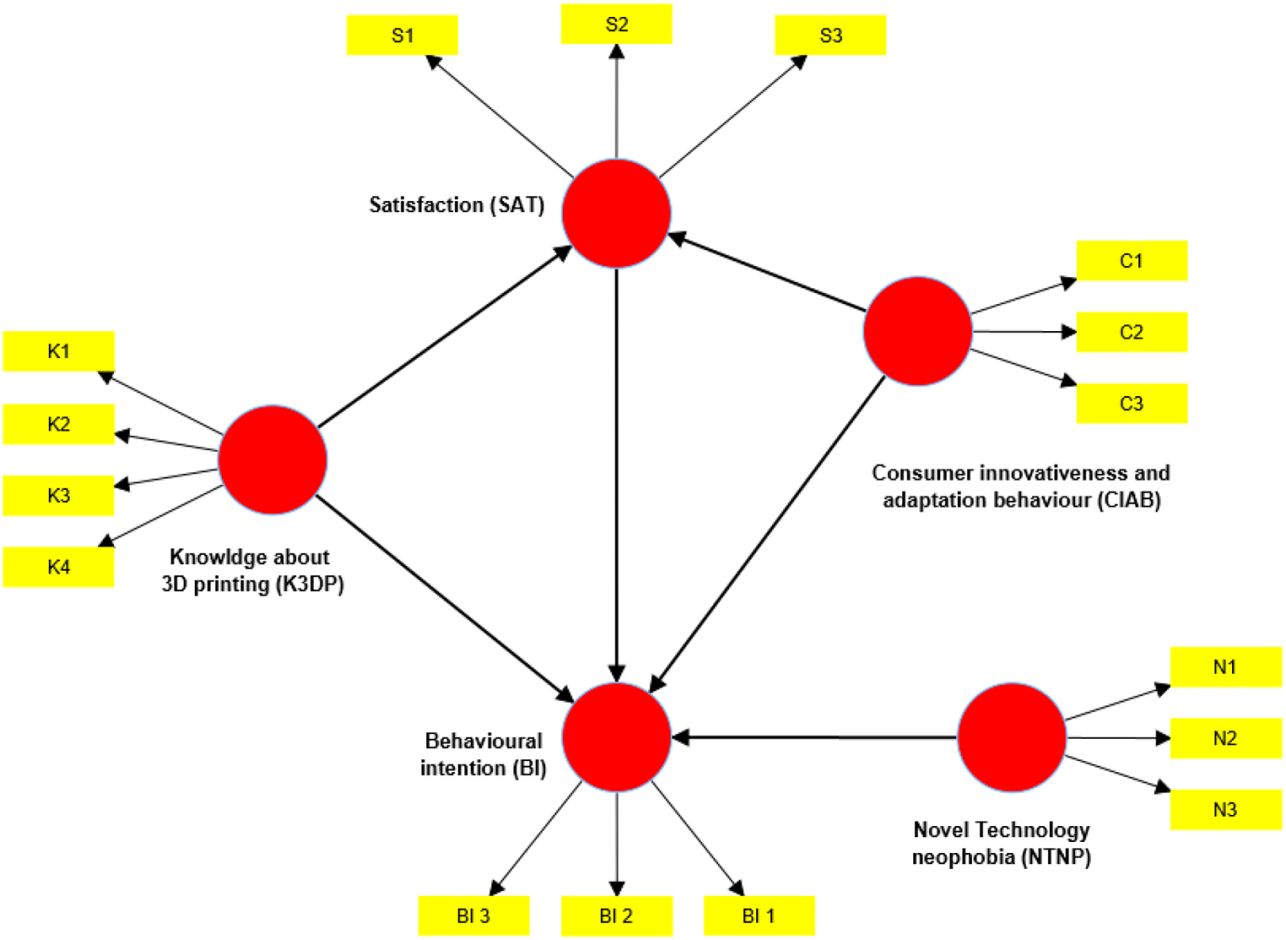
The conceptual model for analysis

A confirmatory composite analysis (CCA) was conducted within the PLS-SEM framework to validate our model. The model achieved a good fit, indicated by a square root mean residual (SRMR) value of 0.066 for the estimated model and 0.065 for the saturated model, well within the acceptable range (< 0.09) as defined by Hair Jr et al. (2014). Additionally, the root mean square error of approximation (RMSEA) stood at 0.06, further confirming the model’s consistency with the data (Fig. 1). Model fit was also evaluated using geodesic discrepancy (d_G_) and unweighted least squares (d_ULS_), yielding values of 0.0226 and 0.451 respectively, suggesting an acceptable fit for structural testing (Hair et al. 2012). Factor loadings (λ), standard deviation (σ), Cronbach’s alpha (α_C_), composite reliability (CR), convergent validity (AVE), and variance inflation factor (VIF) for latent variables (LVs) are detailed in Table 1. Most factor loadings exceeded the 0.7 threshold (at *p* < 0.05, t-statistics above ± 1.96), indicating substantial loading, except for one (N3 in NFTN) which was excluded from subsequent analysis. The model’s reliability was affirmed with all latent variables showing composite reliability (CR) values ranging from 0.7 to 0.93. Convergent validity, assessed using average variance extracted (AVE), was above the 0.5 benchmark for all but one latent variable (SAT, with AVE = 0.421). However, SAT was retained, drawing on research by Acquila-Natale and Iglesias-Pradas (2020) supporting AVE values above 0.4 with CR greater than 0.6. Multicollinearity was not an issue as both inner and outer variance inflation factor (VIF) values were below 5. Discriminant validity was confirmed as average variance extracted for each latent variable exceeded their inter-construct correlations (Table S2). Thus, the model demonstrated reliable measures with both convergent and discriminant validity, effectively controlling for common methods bias.

**Table 1:** Description of our questionnaire’s measuring items and accuracy analysis of the latent variables

Latent variables and their constructs	λ	α_C_	σ	CR	AVE	VIF
Knowledge on 3D food printing		0.82	0.459	0.89	0.581	
K1: *Yes*,* I am familiar with the term “3D food printing*,*” and I understand that it is used for the production of delicious foods.*	0.933					1.016
K2: *I have heard/read and understand about 3D food printing*	0.731					1.023
K3: *I have seen a 3D food printer working*	0.855					1.059
K4: *Have you ever wondered what they put in food to make it 3D-printable and how they do it*	0.747					1.038
Novel food technology neophobia (Cox and Evans 2008)		0.76	0.426	0.81	0.504	
N1: *High-tech food items are unnecessary since the sweets I consume now are perfectly enough*	0.974					1.018
N2: *3D printed sweets employing new technology are not healthier than sweets manufactured using traditional ways*	0.832					1.027
N3: *No*,* there is no need to personalize Indian milk sweets because it does not go to waste.*	0.618					1.002
N4: *I wouldn’t eat 3D-printed food because the technology is still new*,* and the risks seem to be greater than the benefits*	0.768					1.029
Consumer innovativeness and adaptation behavior (Ross et al. 2022).		0.79	0.456	0.93	0.577	
C1: *Personally*,* I think 3D-printed milk sweets would be a great way to cut down on food waste because it can be personalized as the customer wants it.*	0.700					1.221
C2: *Having access to ready-to-eat*,* personalized sweets to prepare at home is a fantastic concept*,* I use computers often*,* so I can train easily.*	0.841					1.371
C3: *I am eager to buy 3D printed Indian milk sweets that are manufactured to match the demands of each person*,* with the proper quantity of nutrients and also appear aesthetically appealing*	0.777					1.366
Satisfaction (Lee et al. 2018)		0.82	0.430	0.745	0.421	
S1: *I like the idea of personalized 3D printed foods made specifically for an individual’s needs*,* in tolerances*,* preferences*, etc.	0.741					
S2: *Instead of modifying texture of food by hand*,* I’d rather have my sweets printed to optimized texture*,* with the precision afforded by software integration.*	0.848					
S3: *Having access to ready-to-eat*,* personalized sweets to prepare at home is a fantastic concept.*	0.749					
Behavioral intention (Lee et al. 2018)		0.81	0.401	0.85	0.601	
BI1: *The term “3D printed Indian milk sweets” brings to mind of a delicious*,* newly designed*,* and presumably healthful variety of Indian sweets made using technological advancement.*	0.862					1.018
BI2: *3D printing enables for fast and precise manufacturing of foods. I intend to consume 3D printed milk sweets.*	0.874					1.050
BI3: *I intend to use 3D printer to tailor the texture and nutrition of my food to my exact preferences.*	0.727					1.034

*→ p-value < 0.05, λ→ factor loading, α_C_→ Cronbach’s alpha σ→ Standard deviation C.R. → Composite reliability, AVE → Average variance extracted, VIF →Variance inflation factor

### The structural model

Table 2 shows a structural model including gender (GEN), age (AGE), and education level (LOE) as control variables. Results indicate males are more inclined to adopt 3DFP than females, aligning with previous studies assessing consumer attitudes toward emerging technologies in the food industry. Younger adults show more positive attitudes towards 3DFP compared to older ones and those with master’s degrees or higher are likelier to try 3DFP than those with lower educational levels. Post-mediation analysis, these control variables were reintegrated into the structural model.

**Table 2:** Path analysis standardized total effect bootstrapped results

Hypothesis	Coefficient	St. Err.	*p* - values	Hypothesis acceptance
Consumer innovativeness and adaptation behavior (CIAB) -> Behavioral intention (BI)	0.200	0.075	0.003*	Accepted
Consumer innovativeness and adaptation behavior (CIAB) -> Satisfaction (SAT)	0.158	0.071	0.025*	Accepted
Novel Technology neophobia (NTNP) -> Behavioral intention (BI)	-0.024	0.054	0.653	Not accepted
Knowledge about 3D printing (K3DP) -> Behavioral intention (BI)	0.406	0.078	0.000*	Accepted
Knowledge about 3D printing (K3DP) -> Satisfaction (SAT)	0.366	0.079	0.000*	Accepted
Satisfaction (SAT) -> Behavioral intention (BI)	0.216	0.073	0.003*	Accepted

*p < 0.05

Table 3 reveals positive correlations between prior knowledge of 3D food printing, behavioural intentions (BI), consumer innovativeness & adaptability, and satisfaction (β values: 0.485*, 0.233*, and 0.216* respectively), supporting hypotheses H1, H2, and H4. Prior knowledge of 3D food printing also positively relates to satisfaction (β = 0.366*), and consumer innovativeness & adaptability to SAT (β = 0.158*), affirming H5 and H6. However, H3, concerning novel technology neophobia’s effect on BI, was rejected (β = -0.022, not significant). Satisfaction’s mediating role between previous 3D printing knowledge, BI, and consumer innovativeness & adaptability was analyzed following Hair Jr et al. (2014). With novel technology neophobia unrelated to satisfaction, the analysis focused on the other relationships.

**Table 3:** Evaluation of mediators and indirect effects in the structural model

Hypothesis			Coefficient		St. Err.	*p* - values
Path estimation without mediators						
Consumer innovativeness and adaptation behavior (CIAB) -> Behavioral intention (BI)			0.605		0.070	0.004*
Knowledge about 3D printing (K3DP) -> Behavioral intention (BI)			0.807		0.076	0.000*
Indirect effects						
Consumer innovativeness and adaptation behavior (CIAB) -> Satisfaction (SAT) -> Behavioral intention (BI)			0.34		0.022	0.015*
Knowledge about 3D printing (K3DP)-> Satisfaction (SAT) -> Behavioral intention (BI)			0.79		0.033	0.017*

Mediation via SAT	Direct effect without mediator	Indirect effect	Direct effect with the mediator	VAF	Mediating role of satisfaction	
Consumer innovativeness and adaptation behavior (CIAB) -> Behavioral intention (BI)	0.605	0.34	0.233	0.593	Partial	
Knowledge about 3D printing (K3DP) -> Behavioral intention (BI)	0.807	0.79	0.485	0.619	Partial	

Notes: VAF = indirect effect/total effect. VAF < 0.20:no mediation; 0.20 < VAF < 0.80:partial mediation; VAF > 0.8:full mediation (Hair et al. 2014)

The mediation analysis used a two-stage approach: first, establishing the direct effect’s significance between independent and dependent variables without the mediator, then assessing the mediator’s indirect effect using Eq. 1.

1 $$\:z=\frac{a\mathrm{*}b}{\sqrt{{a}^{2}{{S}_{b}}^{2}+{b}^{2}{{S}_{a}}^{2}}}$$

where, $\:a$ is the path coefficient between the independent latent variables and the mediator, and $\:b$ is the path coefficient between the mediator and the dependent latent variable, $\:{S}_{a}$ represents standard deviation error of path $\:a$, and $\:{S}_{b}$ represents the standard deviation error of path $\:b$.

The significant indirect effects *(p* < 0.05) indicated mediation relevance. Mediation strength was assessed using the variance accounted for (VAF), calculated as (Indirect effect)/(Total effect) Hair Jr et al. (2014). A VAF > 80% implies complete mediation, 20% < VAF < 80% suggests partial mediation, and variance accounted for < 20% indicates no mediation.

The model displayed partial mediation (Table 3), with SAT playing a stronger mediating role in the prior knowledge of 3D food printing– behavioural intentions relationship (VAF = 0.619) than in CIAB-BI (VAF = 0.593). This shows BI’s positive association with prior knowledge of 3D food printing, especially when satisfaction with 3D-printed foods is high. SAT also enhances the positive impact of consumer innovativeness & adaptability on behavioural intentions, suggesting customers open to new foods and technology are likelier to engage when satisfied with the technology’s role, thus confirming hypotheses H7a and H7b.

**Fig. 2:**
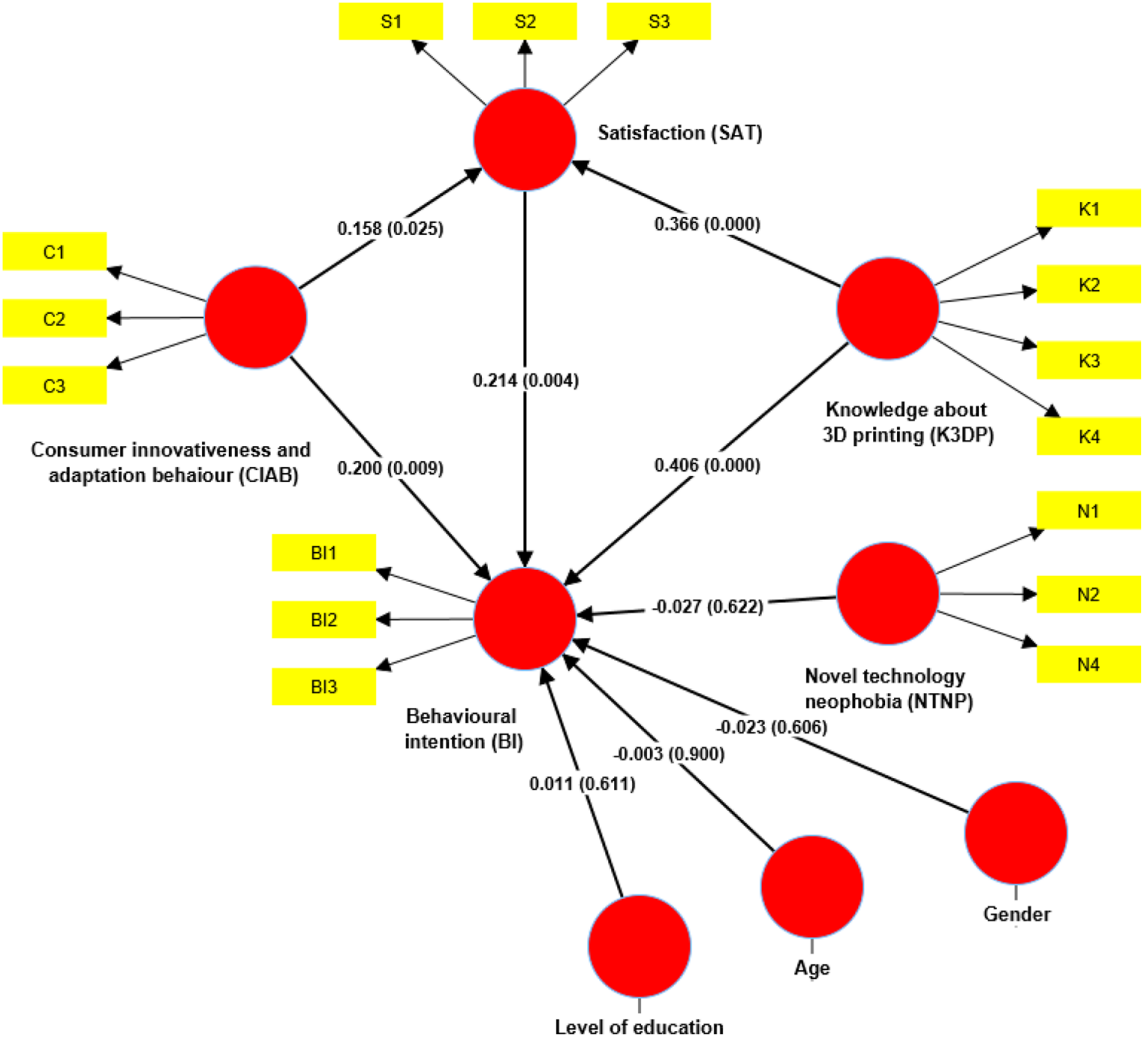
The structural model including path coefficients and their *p* values with control variables

Finally, reintegrating gender, age, and education level into the structural model post-mediation (Fig. 2) showed no significant correlation between these variables and behavioural intentions (β values: -0.03, -0.023, 0.011), nor did they notably alter the model’s mediation outcomes (Table S3). Therefore, these control variables are deemed ineffective in impacting the overall data and analysis.

### Sensory evaluation

**Fig. 3:**
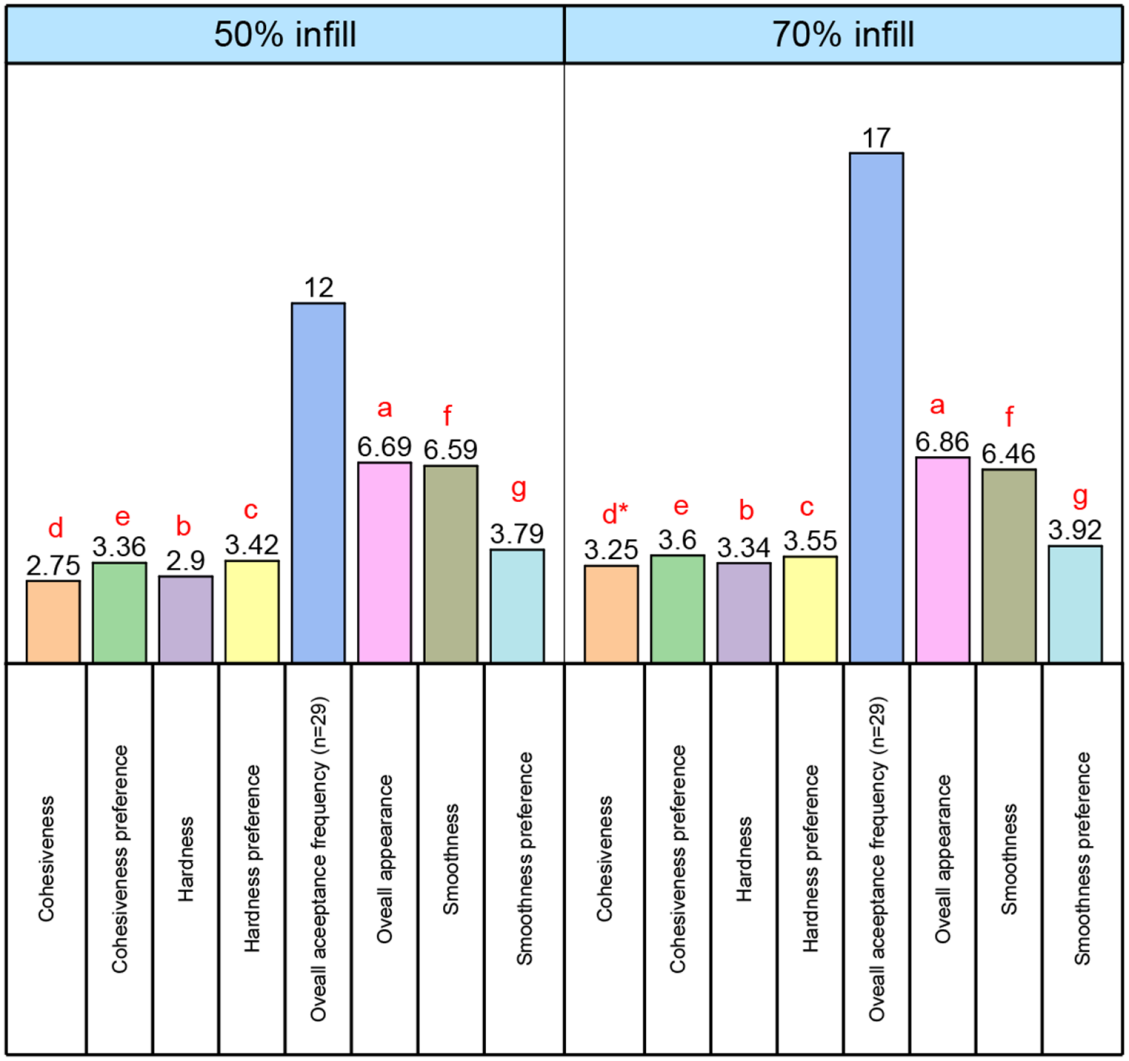
Evaluation of sensory analysis using samples 3D printed with 50% and 70% infill density. Different letters on the plot bars indicate the significant difference (*p*<0.05), according to Scheffe’s test

The sensory evaluation results of 3D-printed with 50% infill and 3D-printed with 70% infill samples, are shown in Fig. 3, including their sensory attributes. The results demonstrate the printed samples, which differ in terms of change in infill density, differ significantly (*p* < 0.05) in cohesiveness. From Fig. 3, it is clear that 70% infill was the preferred choice for 3D-printed HACMP sweets. Also, the 3D-printed sample with 50% infill density scored lower than the 70% infill sample in terms of its overall appearance.

## Discussion

This study makes a primary contribution by employing a simple random sampling approach setting it apart from previous research that often focused on narrower consumer segments. We broaden the scope to encompass the overall behavioral intention of a diverse consumer population. This includes an array of genders, nutritional needs, and operational contexts, moving beyond the limitations of studies restricted to consumers who are experts in 3D food printing (Burke-Shyne et al. 2020), operational contexts (Caulier et al. 2020), or food sector-specific contexts (Kocaman et al. 2023). Although our study specifically targets a subgroup– people who have access to technology– the random sampling approach offers novel insights. In addition, and perhaps more significantly, the findings in this study would help to broaden consumer perception of 3DFP in domestic applications and enhance its predictability by considering motivational (satisfaction), personal (prior knowledge of 3D food printing) and behavioural factors (consumer innovativeness & adaptability) in addition to rational and social ones (novel food technology neophobia), as antecedents to behavioural intentions (BI) of the consumers which have not yet been extensively and separately examined.

### Demographic influence on 3DFP acceptance

Contrary to established beliefs, this study reveals that demographic factors, including gender and education, do not significantly influence consumer acceptance of 3D-printed food. This finding suggests an evolving societal trend where technological advancements in food preparation hold a universal appeal, transcending traditional demographic boundaries. It may reflect a growing digital literacy across different groups or an inherent attraction to the novel culinary experiences offered by 3DFP. It does support the idea that societal divisions have a common origin in diversity. This may be since the benefits in the survey concerned widely consumed homemade delicacies (traditional Indian milk sweets), guaranteeing that customers of all ages and gender would be familiar with the product and, therefore, able to grasp the context of the 3DFP for domestic applications. As Brunner et al. (2018) argued tailored communication significantly impacts customers’ attitudes and assumptions regarding 3DFP. Also, by addressing the knowledge gap identified by Tesikova et al. (2022), this study meets a need for more preliminary empirical data about the applicability of the 3DFP for domestic use.

### Comprehensive factors influencing consumer intent

Our analysis indicates that consumer behavior towards 3DFP is influenced by a blend of rational considerations, such as health benefits, and emotional factors like personal curiosity. This multifaceted approach reveals a market trend where consumers place a high value on unique experiences and personal interaction with their food choices, suggesting that the decision-making process extends beyond mere practicality.

### Importance of knowledge and awareness (K3DP)

The pivotal role of consumer knowledge in the acceptance of 3DFP underscores the necessity for targeted educational initiatives and transparent communication strategies. This insight suggests that dispelling misconceptions and enhancing awareness about the safety and benefits of 3DFP could be key drivers for broader market acceptance.

### Openness to new technologies

The study highlights a strong correlation between the willingness to experiment with new foods and positive attitudes towards 3DFP. This trend might be indicative of a larger cultural shift towards embracing technology in everyday life, positioning 3DFP as a potentially mainstream culinary practice in the near future. Consumers who are optimistic about technology and can see its benefits are more likely to accept it because they are more eager to taste novel foods and less likely to be persuaded by unfavourable impressions. Consumers’ initial hesitations towards 3D-printed foods may be alleviated by the fact that the introduction of 3DFP in the widespread domestic sector empowers them with the freedom and autonomy to experiment with 3D-printed foods with relatively low risk compared to the risks associated with unknown preparation and delivery in the catering and food service industry. 3DFP enables, for instance, the modification of conventional milk sweets in innovative ways, such as by altering their texture and appearance (Bareen et al. 2023). This enables consumers to create personalized sweets that are themed for special occasions without paying exorbitant prices.

### Complex consumer decision-making process

Our findings illustrate that consumer decisions to adopt 3DFP are influenced by a complex interplay of logical assessments and emotional responses. This insight is crucial for developing marketing strategies that not only highlight the practical benefits of 3DFP but also its emotional and experiential appeal.

### Perception overriding technological concerns (NTNP)

The study indicates that concerns about the negative implications of technology (NTNP) have a lesser impact on the decision to use 3DFP. For the sake of this analysis, a food-centric framework of traditionally prepared milk sweets was adopted. Due to the nature of the application, it is possible that participants did not view the resulting 3D-printed food as unprecedented food. Consequently, the participants’ perception of the printed food was more relevant than the innovativeness of the technology itself. This suggests that as long as the final product meets or exceeds expectations in quality and taste, the technological means of production becomes a secondary concern. This insight is vital for formulating product-centric marketing approaches.

### Sensory appeal and consumer acceptance

The results underline the importance of sensory qualities in the acceptance of 3D-printed foods. While the novelty of 3DFP generates initial interest, its long-term market success is contingent upon its ability to meet, if not surpass, the sensory standards of traditional food preparation methods.

## Perspectives and conclusion

The study assumed that 3DFP would be widely available in the near future; however, it is also essential to consider factors such as the cost of 3D food printers and the availability of suitable ingredients. These factors may play a significant role in determining consumer acceptance of 3DFP. Additionally, this study was limited to Indian customers, and cultural and societal conventions vary by country. It would be interesting to study the perspectives of consumers from other regions. The study also relied on self-reported data, which may introduce inaccuracies, such as inconsistency over time and inaccurate estimates, we have taken measures to minimize inaccuracies, such as using validated questionnaires and conducting data triangulation using a confirmatory composite analysis within the PLS-SEM framework. In future studies, directly collected data with a longitudinal approach would be more accurate and allow for examining changes in attitudes and behavior over time. Additionally, the cross-sectional design of this study does not allow for the identification of causality between variables. A prospective study would be needed to explore cause-and-effect relationships between the adoption of 3DFP and related factors.

This study highlighted the importance of understanding consumer perspectives on implementing 3DFP within a domestic setting. As 3DFP technology is not yet widely available to wide consumers, this study aimed to identify critical factors that are important to them. To successfully introduce 3DFP in the home to a broader population, a comprehensive approach is required, addressing factors such as consumer innovativeness and knowledge of the technology through product design and marketing strategies that emphasize the benefits of personalization at home. Consumer acceptance of 3D printers as kitchen appliances may depend on factors such as ease of use and availability of food-grade materials. This research on consumer perception of 3DFP in India is significant as the country is expected to see a substantial increase in global consumption in the 21st century, providing insight into the food-related practices and challenges of Indian consumers.

## Electronic supplementary material

Below is the link to the electronic supplementary material.

Supplementary Material 1

## Data Availability

The datasets used and/or analyzed during the current study are available from the corresponding author on reasonable request.

## Code availability

Not applicable. The manuscript does not contain any such custom code or software application.
