# Supplementary material for: Understanding the intention of consumers towards 3D food printing: exploratory study of psychological factors and sensory analysis
Source: J Food Sci Technol. 2025 Mar 18;63(6):1128–39. doi: 10.1007/s13197-025-06261-8 (PMC13184009; doi:10.1007/s13197-025-06261-8)
Supplement: Supplementary file 1 — Supplementary Material 1 [file 13197_2025_6261_MOESM1_ESM.docx]

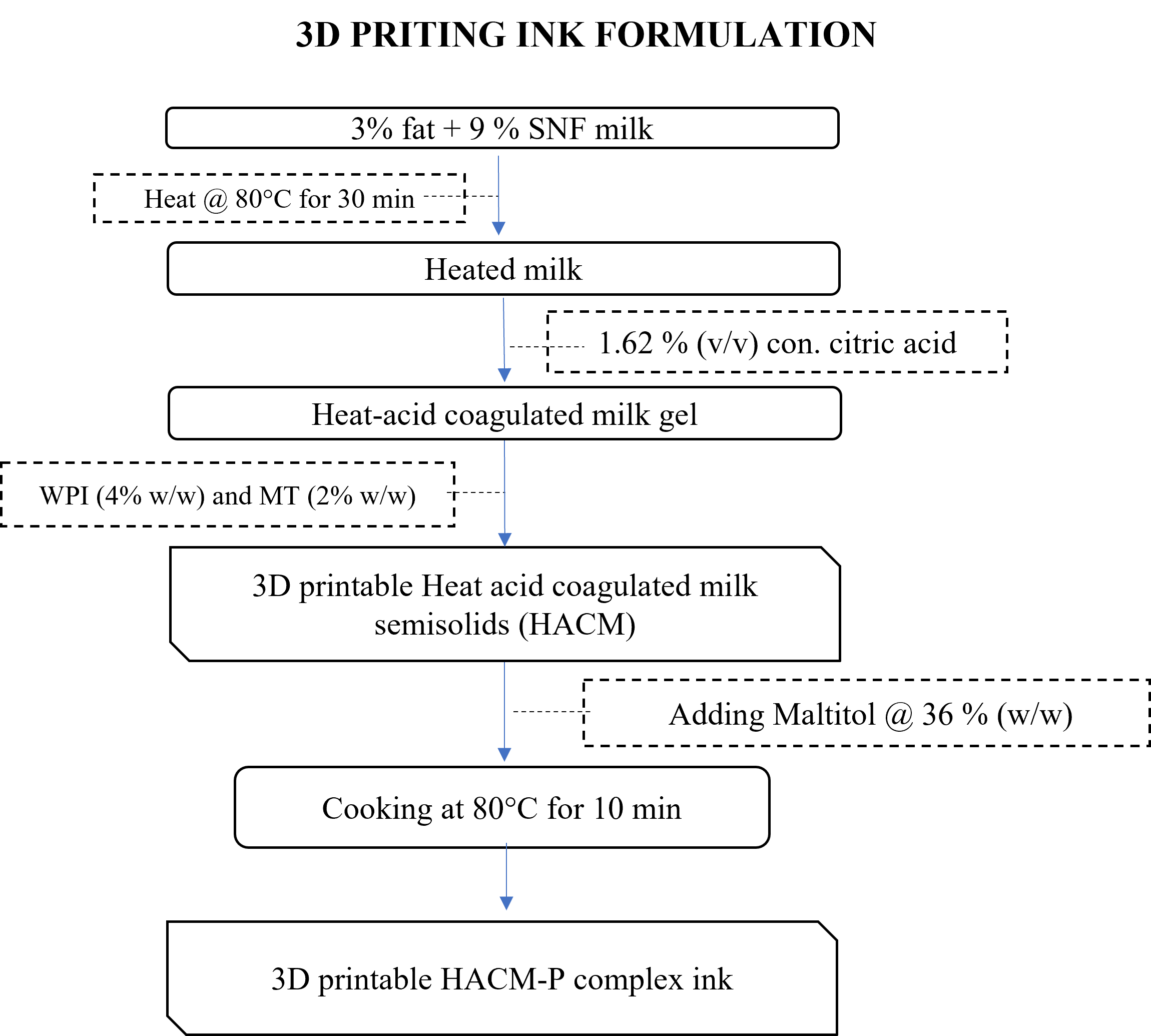


**
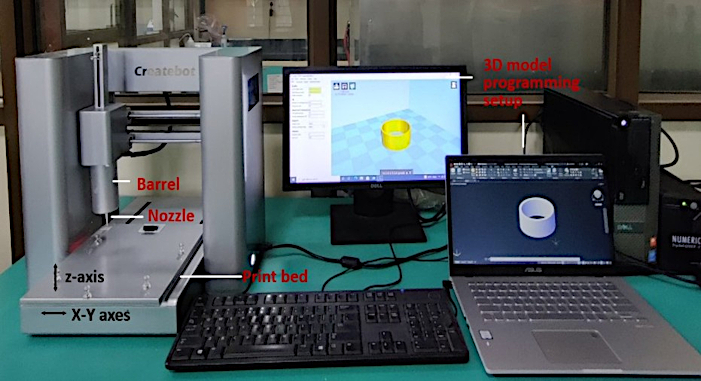
**

**(b)**

**Fig. S1** **(a)** Flow chart of the 3D printing ink formulation process – heat acid coagulated milk semisolid and polyol (HACMP) complex ink, (b) 3D printing set up used for this study.


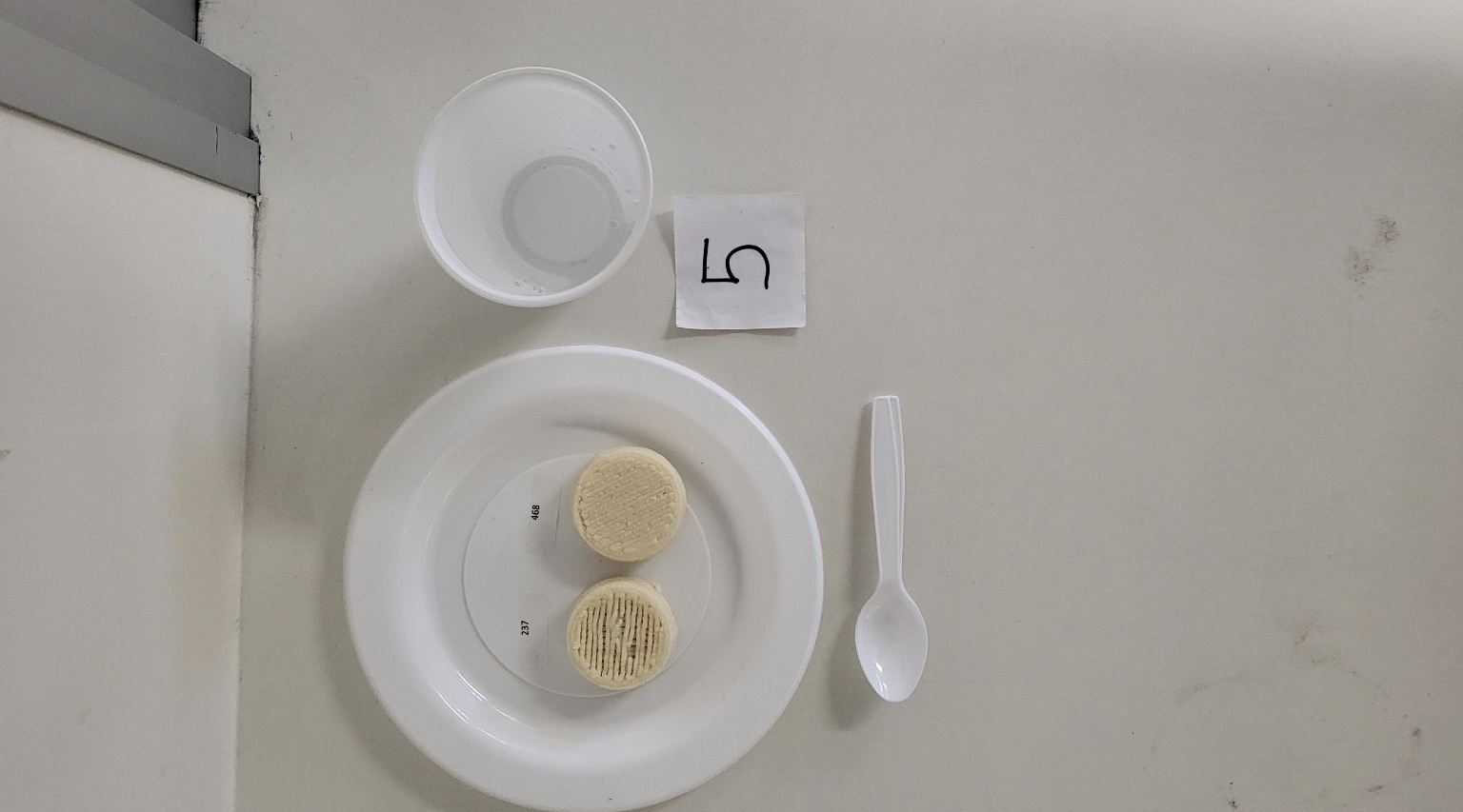


Fig. S2. 3D printed samples one with a 50 % infill (labeled as 237) and one with a 70 % infill (labeled as 468). Each participant is represented by a unique number on a white sheet of paper served with the sample tray at the beginning of the test.

Table S1 Characteristics of the sample population and descriptive statistics.

| Sample characteristics | Number (n=277) | Population percentage (%) |
| --- | --- | --- |
| Gender |  |  |
| *Female* | 84 | 30.4 |
| *Male* | 193 | 69.6 |
| *Subtotal* | 277 | 100 |
| Age |  |  |
| *18-24* | 109 | 10.1 |
| *24-34* | 104 | 81.6 |
| *35-44* | 61 | 7 |
| *45-54* | 3 | 1.3 |
| *Subtotal* |  | 100 |
| Education |  |  |
| *Secondary* | 12 | 4.3 |
| *Bachelors* | 102 | 36.8 |
| *Masters* | 100 | 36.2 |
| *Doctoral* | 63 | 22.7 |
| *Subtotal* |  | 100 |

Table S2 Latent variable correlation and discriminate validity (Fornell–Larcker Criterion).

| Latent variables | 1 | 2 | 3 | 4 | 5 |
| --- | --- | --- | --- | --- | --- |
| 1. Novel food technology neophobia | **0.709** |  |  |  |  |
| 2. Knowledge on 3D food printing | 0.474 | **0.762** |  |  |  |
| 3. Consumer innovativeness and adaptation behavior | 0.233 | 0.206 | **0.759** |  |  |
| 4. Satisfaction | 0.573 | 0.398 | 0.222 | **0.648** |  |
| 5. Behavioral intention | 0.459 | 0.363 | 0.159 | 0.445 | **0.775** |

Diagonal values represent square root of average variance extracted (AVE)

Table S3 Standardized mean scale values for age, gender, education, and latent variables.

| Latent variables | Novel food technology neophobia | Knowledge on 3D food printing | Consumer innovativeness and adaptation behavior | Satisfaction | Behavioral intention |
| --- | --- | --- | --- | --- | --- |
| Mean | 0.712 | 0.696 | 0.760 | 0.791 | 0.708 |
| Gender |  |  |  |  |  |
| *Female* | 0.702 | 0.689 | 0.718 | 0.718 | 0.734 |
| *Male* | 0.717 | 0.697 | 0.778 | 0.823 | 0.697 |
| Age |  |  |  |  |  |
| *18-24* | 0.725 | 0.680 | 0.73 | 0.741 | 0.708 |
| *24-34* | 0.676 | 0.678 | 0.745 | 0.751 | 0.699 |
| *35-44* | 0.729 | 0.705 | 0.797 | 0.891 | 0.666 |
| *45-54* | 0.75 | 0.76 | 0.833 | 0.866 | 0.733 |
| Education |  |  |  |  |  |
| *Secondary* | 0.875 | 0.625 | 0.708 | 0.750 | 0.875 |
| *Bachelors* | 0.682 | 0.684 | 0.766 | 0.814 | 0.666 |
| *Masters* | 0.723 | 0.693 | 0.725 | 0.748 | 0.745 |
| *Doctoral* | 0.710 | 0.723 | 0.814 | 0.830 | 0.698 |
